# Assessing the Impact of COVID-19 on Work-Related Quality of Life through the Lens of Sexual Orientation

**DOI:** 10.3390/bs11050058

**Published:** 2021-04-23

**Authors:** Cindy Mendes, Henrique Pereira

**Affiliations:** 1 Department of Psychology and Education, Faculty of Social and Human Sciences, University of Beira Interior, Pólo IV, 6200-209 Covilhã, Portugal; cindy.mendes@ubi.pt; 2 Research Centre in Sports Sciences, Health Sciences and Human Development (CIDESD), 5001-801 Vila Real, Portugal

**Keywords:** COVID-19, work-related quality of life, sexual orientation

## Abstract

In the face of the COVID-19 pandemic and the exceptional situation that has been experienced on a global scale since 2020, it is essential to assess the impact of COVID-19 in several areas and domains. Therefore, this research seeks to evaluate the impact of COVID-19 on work-related quality of life (WRQoL) in a Portuguese-speaking sample, through the lens of sexual orientation. One thousand, five hundred and seventy-seven individuals participated in this study, of which 1396 (88.5%) self-identified as heterosexual, 95 (6.0%) as gay or lesbian, and 87 (5.5%) as bisexual. Participants responded to the “Work-Related Quality of Life” scale, the “Fear of COVID-19” scale, and the “Negative Impact of COVID-19” scale. Bisexuals scored higher on “Fear of COVID-19” and “Negative Impact of COVID-19” than heterosexual, and gay, or lesbian participants. Differences between sexual orientations for all dimensions of WRQoL were found: heterosexual participants scored higher on general well-being, home–work interface, career satisfaction, working conditions, and lower on stress at work, compared to bisexual, and gay, or lesbian participants. Gay or lesbian participants scored lower than heterosexual and bisexual participants on career satisfaction and working conditions. Sexual orientation, the fear of COVID-19, and the negative impact of COVID-19 were significant predictors of overall WRQoL (explaining 13% of variance). Moderation analysis also showed that sexual orientation is a significant moderator of the association between the fear of COVID-19, the negative impact of COVID-19, and WRQoL. LGB people (especially bisexuals) suffer more severe impacts of COVID-19 and have lower WRQoL than heterosexual people. Inevitably, this has consequences in terms of mental health and overall quality of life for sexual minorities, thus reinforcing the need to adopt inclusive policies in organizations and companies to improve their WRQoL.

## 1. Introduction

Performing a work-related activity is an essential component of one’s sense of identity and self-efficacy [1], affecting subjective perceptions of overall quality of life [2]. It is closely associated with goals, perspectives, and life projects. The attributed importance of having a job is a crucial factor to achieving and maintaining physical and mental health, positive social competencies, life satisfaction, productivity, social status, environment, and social relations, and access to material goods [3], thus the concept of work-related quality of life (WRQoL).

WRQoL has only recently been recognized by companies and organizations as a fundamental aspect of their workers’ well-being and satisfaction [4]. To this extent, organizations that show a particular concern with the WRQoL of their employees tend to demonstrate a more humanized approach, conferring more responsibility, professional autonomy, personal development, and personal growth to their collaborators [4,5]. Therefore, WRQoL can be seen as a multidimensional and dynamic psychological construct [6], directly related to individual and situational characteristics [7], which encompasses a set of worker characteristics and specific aspects of the organizational context [5,8]. Perceived levels of WRQoL are usually related to job satisfaction, safety conditions, organizational climate [1,5], interpersonal relationships in the organization, remuneration [7], autonomy and responsibility, and achievement of results [3,6]. However, they may also be influenced by different psychosocial dynamics in the workplace, such as discrimination against sexual minority workers.

In Portugal, the socio-political inclusion of sexual minorities has been a progressive process, with significant changes in Portuguese legislation regarding equal rights for sexual minority people. To this extent, the approval of a same-sex marriage law in 2011 or the adoption by same-sex couples’ law in 2016 [9,10] place Portugal in an inclusive and integrative position regarding sexual minorities’ rights. However, the social reality is still influenced by a strong conservatism mainly related to the predominantly Catholic religion [11,12], which endorses heteronormativity, disqualifies homosexuality, and favors the manifestation of homophobic attitudes, sexual stigma, prejudice, and discrimination [10], consequently generating significant losses in mental health and quality of life for sexual minorities [13]. Hence, it is clear that there is a contrast between inclusive legislation that has progressed toward the integration of sexual minorities and the conservatism of social reality that undermines its affirmation [14].

This is also the case concerning the Brazilian socio-political context, characterized by a strong conservatism, being one of the world’s countries with the highest rates of hate crimes attributed to homophobia [15]. Sexual minorities in Brazil tend to be trapped in a climate of insecurity, hostility, and violence. Specific laws that defend lesbian, gay, and bisexual (LGB) rights and the criminalization of hate crimes against sexual minorities have not yet been implemented [16]. Thus, the urgency to enforce social policies that defend Brazil’s LGB community’s integrity and respect becomes evident [17]. The presence of heterosexual hegemony as the dominant sexual paradigm in these societies often reduces LGB individuals’ access to essential resources, such as education, career opportunities, and social, medical, and political support [18,19].

In organizational and work contexts, it has been found that belonging to a sexual minority can represent a disadvantage in accessing career opportunities and senior positions, and an increased likelihood of exposure to experiences of discrimination due to their sexual orientation [20]. Sexual orientation affirmation has relevant implications for the organizational and professional experiences of LGB people, leading them to frequently omit to mention their sexual orientation in favor of social acceptance [21], which violates one of the fundamental rights of freedom [19]. These aspects are necessarily associated with negative consequences for self-esteem, professional self-confidence, occupational stress [22], and job satisfaction [6,23,24], causing significant risks to mental health and WRQoL [20]. Sexual diversity in work organizations highlights ethics, organizational justice, equity, diversity policies, job satisfaction, and well-being [25]. Nevertheless, what happens in work organizations is in line with what happens in the socio-political context. Some guidelines defend and accept sexual diversity in organizations; however, these policies may not effectively reduce existing discrimination and prejudice in the workplace [22].

The current worldwide pandemic situation due to the new coronavirus, SARS-CoV-2 or COVID-19, is related to an infectious viral disease that mainly affects the respiratory airways [26]. The pandemic began in China in late 2019 and was rapidly declared a significant public health concern worldwide [27]. The pandemic resulted in a global health crisis, and as of March 2021, around 127 million people have been infected [28]. In Portugal, the first recorded cases emerged in early March 2020. In that month, a state of emergency was declared through a general lockdown as well as the adoption of several measures to contain and prevent the virus’s spread [29]. In Brazil, the first cases were registered in February 2020, and in that same month, the country declared a State of Public Health Emergency of National Importance [30]. Inevitably, this situation has had significant impacts in several social and economic areas, including occupational health and work conditions [19,31,32].

Despite the scarcity of studies on this topic, research shows that in other pandemic situations, such as HIV/AIDS, sexual minorities presented decreased results of WRQoL and more significant risks associated with their mental health [33]. These results may be due to the accumulation of stigmatizing barriers, namely, prejudice concerning the disease and their sexual minority status [34,35]. In the COVID-19 pandemic, like most people, sexual minorities have also suffered from job loss [36,37], and organizational and financial problems during the pandemic [19,38], leading to negative emotional consequences [19], due to stress associated with economic issues, unemployment, social isolation, and low WRQoL [39]. Sexual minorities typically suffer from vulnerabilities and disadvantages in the workplace, which can be exacerbated in an adverse situation such as the COVID-19 pandemic [23,40].

The necessary policies to combat the spread of COVID-19, such as social isolation and lockdown measures, have had relevant implications for work conditions. Understanding how these abrupt emergent changes affected WRQoL is of the utmost importance. For example, COVID-19 has fundamentally changed workplace geographies, with large proportions of people working from home [41]. Nevertheless, the opportunity to do so may be unevenly distributed, and socially disadvantaged groups may not have the ability to work from home if they choose. COVID-19 poses risks and changes for workers, workplaces, and work practices that are likely to result in disparate effects. Therefore, it is necessary to understand the importance of moderating factors, such as sexual orientation, in the aggravation of the impact of COVID-19 on WRQoL. Previous studies have explored the moderation effect of sexual orientation on health-related outcomes, consistently reporting that heterosexual people presented higher protective effects than gay or lesbian and bisexual people [42,43,44], but no studies were found concerning the moderation effect of sexual orientation on WRQoL, especially with Portuguese-speaking samples. Given that the current pandemic situation is still a public health concern in Portuguese-speaking countries, it is vital to give more disadvantaged social groups, such as LGB people, proper attention, and understand the extent to which the pandemic can aggravate existing frailties.

This study aims to assess the impact of COVID-19 on WRQoL, through the lens of sexual orientation. More specifically, the following objectives were posed: (a) to compare differences in WRQoL and the impact of COVID-19 according to sexual orientation; (b) to assess the predictive effects of the independent variables (“Sexual Orientation”, “Fear of COVID-19” and “Negative Impact of COVID-19”) on all six dimensions of WRQoL; and (c) to assess how the fear of COVID-19 and the negative impact of COVID-19 predicting WRQoL is moderated by sexual orientation. To address these objectives, the following hypotheses were posited: (1) sexual minority participants show lower levels of WRQoL than heterosexual participants; (2) sexual minority participants show higher levels of fear of COVID-19 and negative impact of COVID-19 than heterosexual participants; (3) sexual orientation, fear of COVID-19, and negative impact of COVID-19 are significant predictors of WRQoL; and 4) sexual orientation is a significant mediator of the association between the fear of COVID-19 and negative impact of COVID-19, and WRQoL.

## 2. Materials and Methods

### 2.1. Sociodemographic Questionnaire 

We questioned participants about their age, marital status, education, socioeconomic status, professional status, residence, and self-assessment of sexual orientation within three categories (heterosexual, bisexual, and gay or lesbian).

### 2.2. Fear of and Negative Impact of COVID-19

The fear of COVID-19 scale was developed by Ahorsu et al. [45], and encompassed seven items, ranging in score from 1 to 5 as measured by a Likert-type scale, with higher scores meaning a greater fear of COVID-19 [42]. Examples of questions are as follows: “It makes me uncomfortable to think about corona,” “When I watch news and stories about corona on social media, I become nervous or anxious,” or “I am afraid of losing my life because of corona.” The negative impact of the COVID-19 scale allowed measurement of the participants’ perception of the negative impact that the pandemic has had on their lives [46]. It consisted of ten items related to the various psychosocial functioning areas, ranging in score from 1 to 5 as measured by a Likert-type scale, with higher scores meaning the more significant negative impact of COVID-19 [41]. Samples of questions are as follows: “Compared to my life before the COVID-19 pandemic, … had a negative impact ... on my professional or academic life, … on my family life, … on my financial life.” The internal consistency obtained was α = 0.87 for both scales, indicating excellent reliability [47].

### 2.3. Work-Related Quality of Life

This survey comprises 23 items that assess the participants’ perception of their WRQoL in their institution or organization [48], as measured through six psychosocial sub-factors: general well-being (feelings of happiness and satisfaction with life), home–work interface (the relationship and balance between personal and professional life), career satisfaction (level of satisfaction with their career and work), control at work (level of perceived control in the execution of professional tasks in the work environment), working conditions (related to working conditions, safety, and resources that the person has in his/her workplace), and stress at work (related to the level of stress that the person perceives related to his/her work) which was reversely coded. A 24th item, “I am satisfied with the overall quality of my working life,” was included to provide an outcome variable for measuring overall perceptions of WRQoL. Respondents were required to answer the questions on a 5-point Likert-type scale (1—“Strongly disagree”; 5—“Strongly agree”). Internal consistency was excellent (α = 0.92) [47].

### 2.4. Sample

A convenience sample of 1577 Portuguese-speaking participants over 18 years old, with a professional (such as a work contract) or academic (such as university enrollment) status, participated in this study. After testing for homogeneity for sociodemographic characteristics between the Portuguese and Brazilian samples, we decided to consider a single sample in the present study.

Table 1 shows that the majority of participants were Portuguese (N = 1221, 76.8%), women (N = 990, 62.8%), heterosexual (N = 1396, 88.5%), and ranged in age between 18 and 74 years (M_age_ = 33.70, SD = 12.97). Regarding sexual minorities, the sample is composed more of gays or lesbians (N = 95, 6%) than bisexuals (N = 87.5.5%), and the majority of bisexuals identify as women. The majority of participants are employed (N = 774, 49.1%) or studying (N = 418, 26.5%).

We carried out this research through an online webpage between October and December 2020. Participation was voluntary, and participants were referred to a linked website explicitly created for this investigation. The first page of the questionnaire explained the study’s objectives and informed participants about how to fill it in, withdraw from the study, and contact the authors for more information. Participants also read and agree to an informed consent waiver.

We sent about 8000 notifications, and 1577 participants responded voluntarily (19.71% response rate). The survey distribution complied with all of the ethical principles of informed consent, anonymity, and confidentiality. We offered neither rewards nor other incentives. Inclusion criteria included being older than 18 years of age and being a Portuguese native speaker (from Portugal or Brazil). We obtained ethical approval for this study from the Ethics Committee of the University of Beira Interior, Portugal (code CEUBI-Pj-2020-088).

### 2.5. Data Analysis

We performed descriptive statistics to describe the sample (mean, standard deviation, frequencies, and percentages). We conducted one-way ANOVAs to evaluate differences between comparison groups, in this case, between different sexual orientations, to assess the differences in relation to WRQoL, fear of COVID-19, and negative impact of COVID-19. We conducted a Pearson correlation coefficients analysis to assess the association between the fear of COVID-19, the negative impact of COVID-19, and WRQoL. We also conducted a hierarchical linear regression analysis to examine the effects of independent variables (“Sexual Orientation”, ”Fear of COVID-19, and “Negative Impact of COVID-19”) on the dependent variables (WRQoL and respective dimensions). Finally, a moderation regression model was used to test the hypothesized moderation effect, in which sexual orientation was a mediator that interferes with the underlying mechanism of the relationships between the fear of and negative impact of COVID-19, and WRQoL. To avoid type I errors, Bonferroni correction tests were run. All statistical procedures were conducted using the statistical package for social sciences (SPSS—version 26) and PROCESS procedure for SPSS (Version 3.5.3).

## 3. Results

### 3.1. Overall Results for the Fear of COVID-19, the Negative Impact of COVID-19 and WRQoL

Table 2 presents the descriptive statistics for all variables under study (mean, standard deviation, maximum, minimum). In general, the sample scored close to the median, except for the “Fear of COVID-19” variable, which scored slightly below the median, indicating lower levels of fear of COVID-19. As for the dimensions of WRQoL, all scores were above the median (work stress being reversely coded), with slightly higher levels of career satisfaction.

### 3.2. Results for All Variables by Sexual Orientation

Table 3 shows results for all main variables under study by sexual orientation, to assess whether there are differences between sexual orientations for the fear of COVID-19, the negative impact of COVID-19, and all subscales of WRQoL. We found significant differences (*p* < 0.05) for all variables except “work control.” Bisexual participants scored higher on the fear of COVID-19 and the negative impact of COVID-19. Heterosexual participants scored higher on all dimensions of WRQoL. Gay or lesbian participants scored lower than did heterosexual participants, but higher than bisexual participants on “Fear of COVID-19”, “Negative Impact of COVID-19”, and most WRQoL variables, including overall WRQoL.

### 3.3. Multiple Linear Regression Analyses Predicting Sexual Orientation, the Fear of COVID-19, and the Negative Impact of COVID-19 Effects on WRQoL

We also conducted seven multiple linear regression analyses to assess the predictive effects of the independent variables (“Sexual Orientation”, “Fear of COVID-19”, and “Negative Impact of COVID-19”) on all six dimensions of WRQoL and overall WRQoL. With this analysis, we concluded that sexual orientation, the fear of COVID-19, and the negative impact of COVID-19 were significant predictors of overall WRQoL (explaining 13% of variance), general well-being (explaining 15% of variance), career satisfaction (explaining 6% of variance), and work conditions (explaining 7% of variance). The fear of COVID-19 and the negative impact of COVID-19 are significant predictors of the home–work interface (explaining 5% of variance). The “Fear of COVID-19” variable was a significant predictor of work control (explaining 1% of variance) and work stress (explaining 8% of variance). See Table 4 for more detailed information on these results.

### 3.4. Fear of and Negative Impact of COVID-19 Predicting WRQoL as Moderated by Sexual Orientation

**Figure 1:**
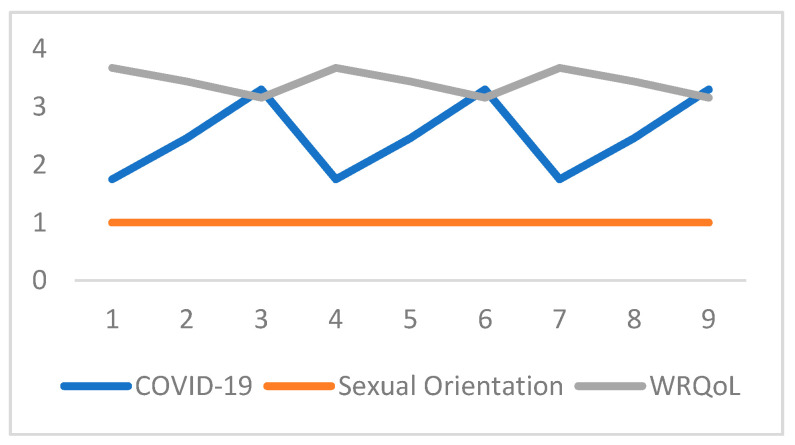
Simple slopes mapping the fear of and negative impact of COVID-19 predicting WRQoL as moderated by sexual orientation. (*p* < 0.001).

Finally, a moderation analysis was performed. Sexual orientation was examined as a moderator of the relationship between the fear of and negative impact of COVID-19 (computed into one single variable—COVID-19) and WRQoL. The model was significant and explained 6% of the decrease in variance in WRQoL (*F*(3;1416) = 28.428; *p* < 0.001, *R^2^* = 0.057). COVID-19 was a significant predictor of WRQoL (*b* = −0.462, *t*(1416) = −6.481, *p* < 0.001) and so was sexual orientation (*b* = −0.398, *t*(1416) = −3.033, *p* = 0.002). The moderation interaction was significant (*b* = 0.131, *t*(1416) = 2.712, *p* = 0.007). Slopes for sexual orientation predicting WRQoL at each level of COVID-19 scores were also significant (b = 0.037, *t*(1416) = −8.921, *p* < 0.001) (Figure 1).

## 4. Discussion

Our study sought to assess the impact of the fear of COVID-19 and the negative impact of COVID-19 on WRQoL, through the lens of sexual orientation. Concerning WRQoL, previous literature has shown significant differences attributable to sexual orientation [49,50,51,52], with manifested lower levels of WRQoL in sexual minorities, and higher burnout levels [52,53]. Our results were similar, since significant differences were found among sexual orientations, favoring heterosexual participants with higher scores for all WRQoL variables over bisexual and gay or lesbian participants. These results may have been mediated by complex heteronormative influences [54], generating frequent disadvantages imposed by social stigma [55,56], particularly in the context of work, which is still ruled by heterosexism [15]. Work, as one of the primary and most central areas of an individual’s life, is one of the places where there has been frequent discrimination and exclusion for sexual minority people [12,57], through marginalization, [23] prejudice, and stigma [12,52,54]. Inevitably, an unfavorable work environment has consequences for the perception of WRQoL in sexual minorities, negatively impacting career satisfaction and general well-being [50], increasing levels of stress at work [51] and negative emotions [54]. In turn, having low WRQoL leads to more significant impairment of mental health [55,56], especially symptoms of anxiety and depression [50,52,55,57].

Other studies [51,58] concluded that an organizational climate of incivility, hostility, discrimination, and exclusion toward LGB individuals generates lower levels of well-being, more stress at work, and burnout, which, in turn, can lead to reduced career satisfaction [54]. These results are congruent with the data found in our research, since bisexual and gay or lesbian participants scored lower in all dimensions of WRQoL. Furthermore, bisexual participants scored lower in general well-being, the home–work interface, and overall WRQoL. They scored higher in stress at work, whereas gay and lesbian participants scored lower in career satisfaction and working conditions. These results are in line with other findings [59], in which bisexuals, compared to gays and lesbians, tend to suffer from sexual identity pressure, since most societies defend a dichotomy of sexual orientation between heterosexual and gay or lesbian, leaving bisexuals at the margin of this binomial script, being more marginalized due to biphobia [50]. In our research, bisexuals scored lower in overall WRQoL, possibly because of more fragile mental health functioning [56,59] associated with feelings of exclusion from heterosexual and gay or lesbian groups [60], reporting lower levels of overall well-being [59].

Recent studies have shown that COVID-19 has psychosocially impacted the general population [24,61,62], namely, at health, economic, political, and social levels [63]. In our study, the impact of COVID-19 was measured through the “Fear of COVID-19” and perceived “Negative Impact of COVID-19” variables. Fear is pointed out as an essential variable when talking about COVID-19 [64], especially concerning “fear of being infected or of infecting others” [58]. Again, bisexual participants scored the highest, both on the levels of fear of and negative impact of COVID-19, likely because of general factors such as mental health impairment [65,66], but also because of specific factors associated with being a sexual minority through the exacerbation of adversity and vulnerability [61], accentuating the already existent discrepancies between heterosexual and LGB communities [15,67]. Our results are similar to those of Barrientos et al. [61], who measured the psychosocial effects of the COVID-19 pandemic in LGB people and found that there are relevant differences attributable to sexual orientation.

The COVID-19 pandemic has had specific consequences on WRQoL, namely, lower levels of job satisfaction and well-being [68], and higher stress levels [63,69]. Still, our findings showed that the fear of and negative impact of COVID-19 negatively correlated with the dimensions of WRQoL. Sexual orientation, fear of COVID-19, and the negative impact of COVID-19 were also strong predictors of lower WRQoL, explaining 12.3% of overall variance, because sexual minorities have additional concerns about work, finances, and income issues during the pandemic [70]. However, there may be protective factors such as resilience [15], which researchers should consider in future studies. Nevertheless, the COVID-19 pandemic seems to have emphasized the inequalities and disparities already existent in society [67,71,72,73], leading to vulnerability situations that are particularly difficult for bisexual people [72]. Therefore, we can conclude that there are differences in the perception of WRQoL between sexual orientations, with a marked disadvantage for bisexuals, perhaps because the lack of visibility of bisexuality in society is associated with greater vulnerability and susceptibility to the influence of social stigma, particularly biphobia [74]. Furthermore, the internalized bi-negativity and psychological distress felt by these participants may translate into more discrimination and mental health problems [75,76,77] that, in turn, can worsen perceived WRQoL.

This study is not without limitations. A convenience sample collected online does not allow the generalization of results. Because the COVID-19 pandemic is still a recent event, the scarcity of studies in this area and its effects on sexual minorities in the workplace still need further investigation. Although homogeneity tests were performed to obtain a single sample, there may be cultural differences between Portugal and Brazil, which may be mediating our results. It is also important to highlight that the reduced number of LGB participants may constitute another limitation. In future studies, we should include more proportionate categories of sexual orientations. Despite these limitations, we believe that this is an important contribution toward understanding the interactions among all variables studied. In future investigations, longitudinal or qualitative studies are suggested to understand the long-term effects of the negative impact of COVID-19 on the different subscales of WRQoL, and mainly to understand the causes of the differences between heterosexual and sexual minorities. Integrating these results in public sexual inclusion and diversity policies in work organizations would also be relevant.

## 5. Conclusions

Socio-political contexts are changing the traditional mentality in Western societies, but LGB people still suffer more severe impacts of the pandemic and have lower WRQoL than heterosexual people. The resulting consequences for mental health and quality of life for sexual minorities reveal a pressing need to adopt inclusive policies in organizations and companies, to improve the WRQoL of sexual minorities.

## Figures and Tables

**Table 1:** Sociodemographic Characteristics by Sexual Orientation (N = 1577, M_age_ = 33.70, SD = 12.97).

Variable	Categories	Subcategory	N	%
		Sexual Orientation		
Gender	Women		990	62.8
		Heterosexual	919	92.9
		Gay or Lesbian	23	2.3
		Bisexual	48	4.8
	Men		584	37.0
		Heterosexual	482	82.5
		Gay or Lesbian	68	11.6
		Bisexual	34	5.9
	Other		3	0.2
		Heterosexual	0	0.0
		Gay or Lesbian	0	0.0
		Bisexual	3	100
Nationality	Portuguese		1211	76.8
		Heterosexual	1108	91.5
		Gay or Lesbian	57	4.6
		Bisexual	46	3.9
	Brazilian		366	23.2
		Heterosexual	287	78.3
		Gay or Lesbian	38	10.4
		Bisexual	41	11.3
Sexual Orientation	Heterosexual		1395	88.5
	Gay or Lesbian		95	6.0
	Bisexual		87	5.5
Marital Status	Single		894	56.7
		Heterosexual	755	84.5
		Gay or Lesbian	68	7.6
		Bisexual	71	7.9
	Married		410	26.0
		Heterosexual	396	96.7
		Gay or Lesbian	4	1.0
		Bisexual	10	2.3
	De facto Union		166	10.5
		Heterosexual	144	86.9
		Gay or Lesbian	18	10.6
		Bisexual	4	2.5
	Divorced		93	5.9
		Heterosexual	87	93.3
		Gay or Lesbian	3	3.3
		Bisexual	3	3.3
	Widower		14	0.9
		Heterosexual	13	92.3
		Gay or Lesbian	1	7.7
		Bisexual	0	0.0
Professional Status	Unemployed		35	2.2
		Heterosexual	28	79.4
		Gay or Lesbian	3	8.8
		Bisexual	4	11.8
	Student		418	26.5
		Heterosexual	363	86.8
		Gay or Lesbian	16	3.9
		Bisexual	39	9.3
	Employed/Student		185	11.7
		Heterosexual	155	84.0
		Gay or Lesbian	14	7.7
		Bisexual	16	8.3
	Self-Employed		132	8.4
		Heterosexual	118	89.2
		Gay or Lesbian	6	4.6
		Bisexual	8	6.2
	Employed		774	49.1
		Heterosexual	703	90.8
		Gay or Lesbian	53	6.8
		Bisexual	18	2.4
	Retired		22	1.4
		Heterosexual	18	80.0
		Gay or Lesbian	2	10.0
		Bisexual	2	10.0
	Other		11	0.7
		Heterosexual	11	100
		Gay or Lesbian	0	0.0
		Bisexual	0	0.0

**Table 2:** Overall results for the fear of COVID-19, negative impact of COVID-19, and WRQoL.

Variables	M	SD	Min	Max
Fear of COVID-19	2.45	0.84	1.00	5.00
Negative Impact of COVID-19	2.60	0.88	1.00	5.00
General Well-being	3.37	0.83	1.00	5.00
Home–Work Interface	3.48	0.91	1.00	5.00
Career Satisfaction	3.60	0.69	1.00	5.00
Work Control	3.43	0.79	1.00	5.00
Work Conditions	3.50	0.85	1.00	5.00
Work Stress	2.90	1.00	1.00	5.00
Overall WRQoL	3.40	0.96	1.00	5.00

**Table 3:** Results for all variables by sexual orientation.

	Heterosexual	Bisexual	Gay or Lesbian		
Variables	M (SD)	M (SD)	M (SD)	*F*	*p*
Fear of COVID-19	2.42 (0.83)	2.71 (0.83)	2.58 (0.94)	6.014	0.003 *
Negative Impact of COVID-19	2.55 (0.86)	3.01 (0.94)	2.98 (0.98)	19.282	0.000 **
General Well-being	3.41 (0.82)	2.98 (0.82)	3.23 (0.87)	12.078	0.000 **
Home–work Interface	3.50 (0.90)	3.25 (0.85)	3.36 (1.02)	3.737	0.024 *
Career Satisfaction	3.62 (0.69)	3.43 (0.67)	3.41 (0.77)	6.418	0.002 *
Work Control	3.43 (0.78)	3.39 (0.81)	3.31 (0.85)	1.081	0.339
Work Conditions	3.53 (0.84)	3.29 (0.87)	3.26 (0.96)	6.591	0.001 *
Work Stress	2.94 (0.98)	2.63 (1.04)	2.76 (1.15)	4.638	0.010 *
Overall WRQoL	3.42 (0.96)	3.16 (0.96)	3.25 (1.07)	3.970	0.019 *

* *p* < 0.05; ** *p* < 0.001.

**Table 4:** Multiple linear regression analyses predicting sexual orientation, fear of COVID-19, and negative impact of COVID-19 effects on WRQoL.

	Sexual Orientation			Fear of COVID-19			Negative Impact of COVID-19				
	*B*	*SEB*	*β*	*B*	*SEB*	*β*	*B*	*SEB*	*β*	*R* ^2^	*F*
General Well-being	−0.094	0.033	−0.069 *	−0.096	0.027	−0.096 **	−0.307	0.026	−0.322 **	0.149	84.769 **
Home–Work Interface	−0.057	0.038	−0.039	−0.072	0.030	−0.067 *	−0.181	0.029	−0.176 **	0.048	24.323 **
Career Satisfaction	−0.056	0.029	−0.050 *	−0.044	0.023	−0.053 *	−0.157	0.022	−0.199 **	0.056	28.907 **
Work Control	−0.034	0.034	−0.027	−0.053	0.027	−0.057 *	−0.006	0.026	−0.007	0.005	2.185
Work Conditions	−0.074	0.035	−0.053 *	−0.117	0.028	−0.116 **	−0.180	0.027	−0.187 **	0.072	36.777 **
Work Stress	−0.003	0.041	−0.002	−0.073	0.033	−0.061 *	−0.282	0.032	−0.250	0.079	40.974 **
Overall WRQoL	−0.058	0.029	−0.050 *	−0.078	0.020	−0.109 **	−0.197	0.019	−0.285 **	0.126	69.112 **

* *p* < 0.05. ** *p* < 0.001.

## Data Availability

The data presented in this study are available upon request.
